# Disinhibition, an emerging pharmacology of learning and memory

**DOI:** 10.12688/f1000research.9947.1

**Published:** 2017-02-03

**Authors:** Hanns Möhler, Uwe Rudolph

**Affiliations:** 1 Institute of Pharmacology, University of Zurich, Zurich, Switzerland; 2 Department of Chemistry and Applied Biosciences, Swiss Federal Institute of Technology Zurich, Zurich, Switzerland; 3 Laboratory of Genetic Neuropharmacology, McLean Hospital, Belmont, MA, USA; 4 Department of Psychiatry, Harvard Medical School, Boston, MA, USA

**Keywords:** Pavlovian learning, disinhibition, somatostatin, GABAA, allosteric modulators

## Abstract

Learning and memory are dependent on interactive excitatory and inhibitory mechanisms. In this review, we discuss a mechanism called disinhibition, which is the release of an inhibitory constraint that effectively results in an increased activity in the target neurons (for example, principal or projection neurons). We focus on discussing the role of disinhibition in learning and memory at a basic level and in disease models with cognitive deficits and highlight a strategy to reverse cognitive deficits caused by excess inhibition, through disinhibition of α5-containing GABA _A_ receptors mediating tonic inhibition in the hippocampus, based on subtype-selective negative allosteric modulators as a novel class of drugs.

## Introduction

Cognitive disabilities are abundant in brain disorders. Apart from a loss of neurons in neurodegenerative diseases and various neurological states, the cognitive deficits are frequently attributed to dysfunctional operations of microcircuits and neuronal networks. This is thought to be particularly the case in neurodevelopmental disorders such as autism spectrum disorders (ASDs) or Down syndrome (DS), in psychiatric disorders such as schizophrenia, but also in age-related cognitive decline. In line with this view, research on cognitive behavior has focused on the role of microcircuits.

Disinhibition was recognized in recent years as an emerging general mechanism of learning and memory. Memories are acquired and encoded within large-scale neuronal networks spanning different brain areas with the respective information being processed by projection neurons in a tightly controlled balance of excitation and inhibition. Recent findings demonstrate that salient events, such as a footshock in aversive learning, often elicit disinhibition of projection neurons (that is, “a selective and transient reduction of synaptic inhibition received by projection neurons that changes their computation” ^1^). The ensuing stronger firing of the projection neuron is likely to gate the induction of synaptic plasticity, required for memory formation. Disinhibition is displayed in different compartments of projection neurons, in diverse cortical areas, and on time scales ranging from milliseconds to days. Behavioral functions of disinhibition range from critical period plasticity ^2^, addiction ^3^, and Pavlovian learning to spatial navigation ^1^. In this article, we will explore the evidence suggesting that disinhibition could become a therapeutic principle for reversal of disease-related cognitive deficits.

## Disinhibition in behaving animals

**Figure 1.**
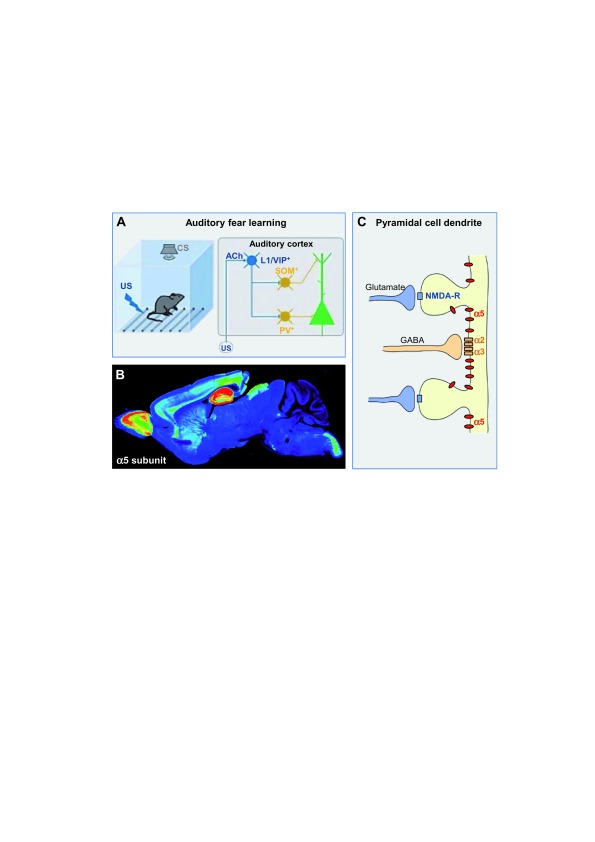
Targets of disinhibition. ( **A**) Disinhibition refers to the selective and transient reduction of synaptic inhibition of a projection neuron due to suppression of interneuron firing by another population of interneurons. A circuit model is given for the cortical disinhibition elicited by an unconditioned stimulus (US) (footshock) in auditory Pavlovian fear learning (modified from 1). Blue color denotes the source of disinhibition, yellow color the inhibited interneurons, and green color the disinhibited pyramidal neurons. Flat bars denote inhibitory inputs. ACh, acetylcholine; CS, conditioned stimulus; PV, parvalbumin; VIP, vasoactive intestinal polypeptide. ( **B**) Immunohistochemical distribution of the α5 subunit of GABA _A_ receptors in mouse brain with false color coding. High expression in hippocampus and cortical layer 5 is shown. ( **C**) Scheme of the extrasynaptic localization of α5 GABA _A_ receptors on dendrites and dendritic spines of hippocampal pyramidal cells, representing the initial, tonic inhibitory control to incoming excitatory signals. Figure 1B provided courtesy of Jean-Marc Fritschy.

Research on disinhibition in behaving animals so far has been strongly focused on aversive learning by fear conditioning, a type of Pavlovian learning. During the acquisition of auditory fear conditioning, disinhibition plays a role in cortical auditory plasticity, which depends on the convergence of activity evoked by both an unconditioned stimulus (for example, footshock) and a conditioned stimulus (for example, tone). Footshocks, which drive learning in this paradigm, elicit strong firing in nearly all layer 1 vasoactive intestinal polypeptide (VIP) interneurons of the auditory cortex, driven by acetylcholine released from basal forebrain afferents. The enhanced activity of inhibitory layer 1 VIP interneurons inhibits layer 1 somatostatin (SOM)-containing GABAergic interneurons, which target the dendrites of principal cells, and parvalbumin (PV)-containing GABAergic interneurons in layer 2/3, which target the soma. With this disinhibitory circuit, the footshock stimulus results in a reduction of inhibition over the entire somatodendritic domain ( Figure 1A). The firing of the cortical projection neurons is enhanced ^4^.

Disinhibition is also recruited by implementing the conditioned tone stimulus as apparent in the amygdala. Principal cells in the basolateral amygdala (BLA) are under the control of inhibitory GABAergic interneurons of which those containing SOM target the dendrites and those containing PV target the soma. During an auditory cue, PV interneurons are excited and indirectly disinhibit the dendrites of the BLA principal cells via SOM interneurons. Thus, activation of PV interneurons by the tone leads to a disinhibition of projection cell dendrites, the site where auditory inputs arrive. Importantly, the dendritic disinhibition overcomes the increases in perisomatic inhibition mediated by excited PV interneurons during the tone, which is the crucial factor in enhancing projection cell tone responses and promoting cue-shock associations in fear learning ^5^.

In addition to its role in memory acquisition, disinhibition is a factor in memory expression. In auditory fear-conditioned mice, the presentation of a conditioned tone stimulus causes strong phasic inhibition associated with inhibition of a subset of PV-positive interneurons in the dorso-medial prefrontal cortex, selectively when mice display a fear reaction. The ensuing disinhibition of projection neurons is required for memory expression. Indeed, in naïve animals, optogenetic inhibition of the corresponding PV cells proved sufficient to induce freezing, indicating that this form of disinhibition is both necessary and sufficient for memory expression. Disinhibition permits stronger responses of the projection neurons to tones that likely drive fear expression in downstream areas such as the amygdala ^1^. Finally, the behavioral motor response of freezing is also triggered by disinhibition. An inhibitory pathway from the central nucleus of the amygdala to the periaqueductal gray (PAG) produces freezing by disinhibition of the excitatory PAG output to the pre-motor targets in the medulla ^6^.

## Genetic disinhibition: downregulation of α5 GABA _A_ receptors enhances learning and memory

To exploit disinhibition pharmacologically, circuits associated selectively with learning and memory would have to be targeted. Starting in 2002, the α5 GABA _A_ receptor subtype (defined as GABA _A_ receptors containing the α5 subunit and β and γ subunits) took center stage ( Figure 1B, C). It is mainly expressed in hippocampal pyramidal cells and layer 5 pyramidal cell dendrites, is located extrasynaptically, generates tonic inhibition, and is capable of altering neural oscillations, which is considered to impact on cognitive behavior ^7– 9^. In genetically modified mice, a partial knockdown of α5 receptors improved hippocampus-dependent performance, as shown in trace fear conditioning ^10^ and appetitive conditioning ^11^, and a full knockdown in α5 knockout mice resulted in improved performance in the water maze ^12^ and improved novel object recognition ^13^, while hippocampus-independent learning was unaltered in both partial and full α5 knockout mice ^10, 13^. Furthermore, although low-frequency stimulation elicited long-term depression in hippocampal slices of normal mice, the same stimulation elicited long-term potentiation (LTP) in slices from α5 knockout mice, suggesting that the receptor sets the threshold for eliciting LTP and contributes to memory formation ^13^. These results make α5 GABA _A_ receptors a key target for a pharmacological enhancement of hippocampus-dependent learning and memory ^14– 16^.

However, the role of α5 GABA _A_ receptors in memory formation may not be as straightforward as described above but depends on the cognitive domain and the context and demand of the task. These findings are a cautionary note on α5 GABA _A_ receptors as targets for α5-negative allosteric modulators (α5-NAMs). For instance, α5 knockout mice exhibited a deficit in short-term memory but only when a particular task (puzzle box) became progressively more difficult, whereas long-term memory deficits were not affected ^17^. A deficit in learning selectivity was apparent in mice with a reduced α5 expression as shown by a deficit in latent inhibition ^18^ and prepulse inhibition ^19^. The memory for location of objects was impaired in these mice, when tested in a complex setting ^20^.

In a conditional knockdown of *Gabra5* selectively in dentate gyrus, performance was impaired in tasks characterized by high memory interference. Such tasks included behavioral pattern separation, when mice had to distinguish between an aversive context and a similar safe context, reversal learning in the Morris water maze, fear extinction, and latent inhibition to conditioned freezing ^21^. In contrast, in tasks characterized by low memory interference (for example, novel object recognition), initial spatial learning in the Morris water maze and fear conditioning to tone, performance was unaltered ^21^. In brain development, a heterozygous α5 knockout, as well as single-cell deletion of α5 in newborn granule cells, caused severe alterations of migration and dendrite development in the dentate gyrus, indicating that relatively minor imbalances in α5 GABA _A_ receptor-mediated transmission may have major consequences for neuronal plasticity ^22^.

## Negative allosteric modulators of α5 GABA _A_ receptors: a new class of drugs

In view of the memory-enhancing findings in the genetically modified mouse models, partial NAMs acting at the benzodiazepine site of α5 GABA _A_ receptors were expected to improve performance in learning and memory. The first ligands of this type were α5IA, L-655,708, and MRK-016 synthesized by the Merck group. The α5IA was selective for α5 receptors only by efficacy and showed equal affinity for α1, α2, α3, and α5 receptors. It improved spatial learning without being anxiogenic or proconvulsant. In elderly volunteers, paired associate learning was enhanced but remained inconsistent. Renal toxicity prevented further development ^23, 24^. L-655,708 showed preferential affinity for α5 GABA _A_ receptors but acted as NAM not only at α5 but also, at higher concentration, at α1, α2, and α3 receptors. It enhanced spatial learning and induced gamma oscillations in hippocampal slices without being proconvulsant, but its anxiogenic effect, presumably due to interactions with receptors other than α5, prevented further development ^24^. MRK-016, like L-655,708, showed preferential affinity for α5 receptors and displayed nootropic properties (for a review, see 14). Most interestingly, disinhibition by L-655,708 and MRK-016 was recently found to produce rapid (measured 24 hours after drug administration), sustained ketamine-like antidepressant effects in animal models of depression, which is likely due to disinhibition-induced plasticity ^25^. Motor recovery after stroke was also enhanced by L-655,708 after chronic dosing (42 days), although the infarct size was unaltered ^26^. Memory in young but not aged rats was improved by the α5-NAM TB21007 ^27^. The α5-NAM PWZ-029 largely lacks functional selectivity, being a weak positive allosteric modulator (PAM) at α1, α2, and α3 GABA _A_ receptors ^28, 29^. In a task assessing working memory, it slightly increased delayed matching-to-sample (DMTS) accuracy in rhesus monkeys, which is likely due to its action as α5-NAM ^29^.

The imidazo-triazolo-benzodiazepine RO4938581 was the first partial α5-NAM with a highly selective preference in both affinity (up to 40-fold) and efficacy (10-fold) for α5 GABA _A_ receptors versus α1, α2, and α3 GABA receptors ^30, 31^. This compound rescued deficits in working memory and spatial memory ^30^ and improved executive functions in an object retrieval task in cynomolgus monkeys ^30^. Most importantly, RO4938581 showed no anxiogenic or pro-convulsive activity. Such agents hold the promise of novel treatments for neurological disorders with cognitive dysfunctions such as DS and cognitive impairment in psychiatric conditions.

## Effectiveness of α5-NAMs in a model of Down syndrome

The best characterized animal model of DS (trisomy 21) is the Ts65Dn mouse, which contains an extra segment of the ortholog mouse chromosome 16 ^32– 35^. The neuronal plasticity in Ts65Dn mice is thought to be obstructed by excessive GABAergic inhibition as low-dose pentylenetetrazole, a non-selective GABA antagonist, reversed deficits in learning and memory ^36^. Remarkably, reducing α5 GABA _A_ receptor function by the NAM α _5_IA was likewise sufficient to reverse the deficits in spatial reference learning and novel object recognition ^37^. The α5-NAM RO4938581 also rescued the spatial performance of Ts65Dn mice, improved the neurogenesis in the dentate gyrus, and normalized the enhanced density of hippocampal GABAergic boutons ^38– 40^.

Clinical trials with RG1662 (Basmisanil), a compound related to RO4938581, were initiated by Roche to improve cognitive disabilities in adults, adolescents, and children with DS ( www.clinicaltrials.gov; Drug RG1662). The phase II trials were recently terminated due to lack of efficacy (Roche Press Release, June 28, 2016; http://www.roche.com/media/store/statements.htm). This outcome suggests either that the α5-NAM was insufficiently effective to restore neuronal plasticity or alternatively that the hypothesis of excessive GABAergic inhibition obstructing neuronal plasticity does not extend to individuals with DS.

## Schizophrenia: cognitive deficits and α5-NAMs

Cognitive deficits are a core symptom of schizophrenia. They are disabling and difficult to treat. Cortical disinhibition has been proposed as an underlying pathological hallmark ^41^. Indeed, in post-mortem brain, a striking deficit of GABAergic inhibition is apparent with several distinct cortical GABA interneurons being dysfunctional (basket cells, Chandelier neurons, SOM/NPY, or CCK interneurons) concomitant with a deficit of GAD67 and a change in GABA _A_ receptor expression, in particular, an increased α2 subunit expression on the axon initial segment ^42^. A deficit in the excitatory drive to cortical PV interneurons is thought to contribute to altered gamma oscillations and cognitive dysfunctions ^43^ as well as to negative symptoms ^44^. GABAergic therapeutic attempts to improve cognitive deficits focused initially on MK-0777, a ligand enhancing synaptic α2 and α3 receptor function ^45, 46^. More recently, enhancing α5 receptor function was found to improve behavioral deficits, at least in the rat methylazoxymethanol (MAM) model of neurodevelopmental disorders, which is characterized largely by a reduction in PV interneurons. The α5-PAM SH-053-2’F-R-CH3 ^28^—when administered either systemically or directly into the ventral hippocampus—reversed the hyperactivation of the dopaminergic system in the ventral tegmental area and dampened the amphetamine-induced increase in the locomotor response ^47^, although not under conditions of haloperidol withdrawal ^48^. It remains to be seen whether a selective α5-PAM would impact on cognitive deficits.

On the other hand, cognitive deficits in patients with schizophrenia are frequently attributed to their inability to recruit high-frequency oscillations while performing cognitive tasks ^49^. Thus, disinhibition of principal cell activity with an α5-NAM might be considered beneficial. In hippocampal slices, high-frequency oscillations were induced by applying the α5-NAM L-655,708 ^50^. Thus, it appears warranted to test whether selective α5-NAMs are able to restore high-frequency oscillations and to ameliorate cognitive deficits in patients with schizophrenia.

## Antidepressant action via α5 GABA _A_ receptors?

The GABA hypothesis of depression posits that a deficit in GABAergic inhibition of principal cells contributes to depression-like behavior ^51, 52^. In this view, an increased inhibitory input to principal neurons might be desirable for circuit-based antidepressant activity. Indeed, a preliminary report indicates that the α5-PAM SH-053-2’F-R-CH3 ^28^ displayed rapid antidepressant-like effects in female (but not male) mice exposed to unpredictable chronic mild stress ^53^. However, contrary to this view, a negative modulation of α5 GABA _A_ receptor with the α5-NAM L-655,708 was recently shown to restore excitatory synaptic strength and to display rapid and sustained antidepressant-like actions after chronic stress in rats ^25^. The stress-induced anhedonia (sucrose preference test) and the deficit in social interactions (interaction test) were reversed by a single dose of L-655,708, measured 24 hours after administration ^25^. Clearly, the discrepancy in the potential antidepressant pharmacology of α5 GABA _A_ receptor ligands remains to be resolved with regard to animal models and ligand selectivity.

## Genetic evidence for a contribution to anxiety regulation by α5 GABA _A_ receptors

Experiments in mice with partial or complete knockout of the α5 subunit revealed no evidence of an involvement in anxiety regulation ^10, 12^. Nevertheless, recent studies showed that α5 GABA _A_ receptors in the central amygdala, specifically those on PKCδ ^+^ neurons, contribute to anxiety regulation. Both a brain area-specific cre-mediated α5 knockdown and a cell type-specific knockdown of α5 using a short hairpin RNA (shRNA) approach resulted in anxiety-related phenotypes in the elevated plus maze test and increased fear generalization ^54^. Thus, in wild-type animals, an enhanced α5 receptor-mediated inhibition of PKCδ ^+^ neurons would be expected to contribute to an anxiolytic response. In keeping with this view, a global enhancement selectively of α5 GABA _A_ receptor function was anxiolytic. Via a “restriction-of-function” approach in which three out of the four diazepam-sensitive α subunits (α1, α2 and α3), and α5 were rendered diazepam-insensitive by a histidine-to-arginine point mutation, it was shown that positive allosteric modulation by diazepam of α5 GABA _A_ receptors with high molecular specificity resulted in anxiolytic-like effects (elevated plus maze and light/dark choice test) at doses higher than those required for diazepam-induced anxiolysis in wild-type mice that would be sedative in wild-type animals ^55^. Thus, enhancing α5 GABA _A_ receptor function contributes to a reduction of anxiety-like behavior. In this context, it is important to note that a pharmacological reduction of α5 GABA _A_ receptor function by selective α5-NAMs did not influence anxiety behavior, as described above.

## The impaired aging brain: a role for α5-PAM?

Increased hippocampal activation has become recognized as a signature of the aging human brain. Hippocampal activation, largely restricted to the CA3/DG regions, is modestly increased in older subjects but, in patients with mild cognitive impairment, greatly exceeds that of healthy age-matched controls ^56^. Reduction of excess neuronal activity by enhancing α5 GABA _A_ receptor-mediated tonic inhibition could potentially improve symptomatic functions and slow prodromal progression. At present, only a few α5-PAM compounds are available: SH-053-2’F-R-CH3 ^28^, compound 44 ^57^, compound 6 ^58^, and MP-III-022 ^59^. However, when interpreting behavioral responses, it has to be kept in mind that the α5-PAMs display only limited selectivity for α5 GABA _A_ receptors. So far, the α5-PAM compound 44 improved long-term memory of aged rats in the Morris water maze, and the α5-PAM compound 6 improved memory performance of aged rats but not of young rats in a radial arm maze task ^27^. Their impact on cognitive behavior remains to be established. Thus, selective α5-PAMs may warrant further testing on the potential amelioration of age-related memory impairments as they are related to hippocampal overactivity.

## Autism spectrum disorders: amelioration by enhancing GABA _A_ receptor function

In a recent behavioral analysis of α5 knockout mice, some autism-like behavior was apparent. Social contacts as tested in the social proximity assay and the three-chamber social approach test were reduced in male mice. Self-grooming as a measure of stereotypy was increased and performance in the puzzle box, reflecting executive functions, was reduced without changes in locomotion or anxiety level ^17^. In contrast to the behavior reported for the α5 knockout mice, autism-like behavior has not been reported to be induced pharmacologically by partial NAMs acting at α5 GABA _A_ receptors.

On the contrary, the observation by Zurek *et al.* ^17^ is in line with the possibility that drugs that act as PAMs of α5 GABA _A_ receptors may ameliorate autism-like behaviors. Indeed, in ASDs, the combinatorial effects of genetic and environmental variables were postulated to cause an excess of excitation versus inhibition (E/I) within key cortical circuits during critical periods of development ^60– 62^. In post-mortem brains of patients with autism, a reduced expression of α4, α5, and β1 subunits has been observed ^63^. Autistic traits in a broad range of ASD models share a reduction of GABAergic signaling within key cortical microcircuits as a common denominator, frequently apparent as a reduction of PV interneurons in neocortex and impaired oscillations ^64, 65^. Attempts to alleviate the GABAergic deficit in ASDs pharmacologically ^16^ were made in two animal models: the idiopathic BTBR model and the Dravet syndrome. In both cases, extremely low doses of benzodiazepines were found to be beneficial, although in a narrow dose range. Although the type of responsive GABA _A_ receptor remains to be established, these results warrant testing selective α5-PAMs in ASDs.

### The BTBR model

In the BTBR model of ASDs, a dramatic behavioral improvement in cognition and social interaction was observed after low-dose benzodiazepine treatment. Non-sedating doses of clonazepam (0.05 mg/kg) improved cognitive deficits, with memory in a context-dependent fear conditioning paradigm being improved in both the short term (30 minutes) and the long term (24 hours) after training, and spatial learning and memory in the Barnes maze was also improved. Likewise, hyperactivity was significantly reduced, as was stereotyped behavior ^66^. Importantly, deficits in social interactions were also improved by low-dose clobazam (0.05 mg/kg). This effect was GABA _A_ receptor subtype-specific since L-838,417, a partial agonist acting at the benzodiazepine site of α2, α3, and α5 GABA _A_ receptors sparing α1 receptors, was likewise effective ^66^. In contrast, zolpidem, acting preferentially on α _1_ receptors, exacerbated social deficits in BTBR mice. These findings support the notion that rebalancing of GABAergic transmission via α2, α3, or α5 GABA _A_ receptors can improve at least some of the ASD symptoms. It remains to be seen whether selective α5-PAMs are similarly effective and offer a broader dose range than the benzodiazepines used so far.

### Dravet syndrome

Dravet syndrome (DrS), also called severe myoclonic epilepsy of infancy, is an intractable developmental epilepsy syndrome caused by a heterozygous loss-of-function mutation in the *SCNA1* gene encoding the α subunit of the Na _v_1.1 sodium channel. DrS is accompanied by neuropsychiatric comorbidities overlapping with ASDs ^67^. Specific heterozygous deletion of Na _V_1.1 in forebrain GABAergic interneurons is sufficient to recapitulate the autistic-like behavioral and cognitive impairments such as hyperactivity, stereotyped behavior, social interaction deficits, and impaired cognition and spatial memory ^68^. Most remarkably, a very low dose of clonazepam (0.0625 mg/kg), which was neither anxiolytic nor sedative, completely rescued impaired social interaction and cognitive deficits in *Scn1a* ^+/−^ mice ^68^. Clinically, clobazam is used in combination with the cytochrome P450 inhibitor stiripentol in the treatment of DrS in children ^69^. α5-PAMs may offer an alternative approach for treatment, possibly with an improved therapeutic window.

## Conclusions

The α5 GABA _A_ receptors are an exceptional target for an enhancement of learning and memory, as they are expressed largely in the hippocampus. They mediate extrasynaptic, tonic inhibition of principal cells.

Disinhibition of α5 GABA _A_ receptor function by genetic means improved hippocampus-dependent learning and memory in classic behavioral tasks but not in complex tasks involving high memory interference.

A variety of PAMs and NAMs have been developed for the α5 pharmacophore ^70^. Pharmacologically, NAMs acting at α5 GABA _A_ receptors improved cognitive and spatial performance in rodents and non-human primates. Cognitive behavioral deficits in DS Ts65Dn mice were normalized. Highly selective NAMs have reached the stage of clinical development. They will permit an assessment of their therapeutic potential in ameliorating cognitive deficits in neurological and psychiatric conditions, including schizophrenia ^71^.

In contrast, other disease conditions may benefit from an enhancement of α5 GABA _A_ receptors. Hippocampal overactivity is a signature of the age-impaired brain, which may respond to α5-PAM treatment to improve function. Furthermore, autism-like behavior likewise may be ameliorated by α5-PAM. The beneficial effect on stereotypies and cognitive impairments, seen with non-selective GABAergic drugs in two animal models, appears to justify a focus on α5 GABA _A_ receptors.
